# The Iowa State Medical Society

**Published:** 1877-07

**Authors:** 


### THE IOWA STATE MEDICAL SOCIETY

Held its annual meeting at Cedar Rapids, June 6, and 7, 1877. About 150 members were present; valuable papers and discussions occupied the usual share of time. The following are the officers for the forthcoming year:

President, Dr. H. Ristine, Cedar Rapids; First vice-President, J. W. Gustine, Carroll; Second vice-President, L. P. Fitch, Charles City; Secretary, J. F. Kennedy, Des Moines; Assistant Secretary, G. O. Morgridge, West Liberty; Treasurer, G. R. Skinner, Cedar Rapids.

Committee of Arrangements: Dr. S. E. Robinson, West Union; Dr. W. H. Ward, Des Moines; Dr. Honnawalt, Des Moines; G. P. Morgridge, West Liberty; H. C. Huntsman, Oskaloosa.

Trustees: First District, H. T. Cleaver, Keokuk; Second District, H. M. Dean, Muscatine; Third District, B. Mc-

Clure, Dubuque; Fourth District, C. M. Hobby, Iowa City; Fifth District, D. McFarland, Keota; Sixth District, J. D. McClerry, Indianola.

---

The Southern Illinois Medical Association, met at Anna, Ill., June 20, at Usery Opera Hall, at 3 o'clock p. m. A large number were in attendance. Dr. L. Dyer, President, occupied the chair. The Association being called to order, prayer was offered by the Rev. K. W. Vansleve, of the Methodist Episcopal Church, after which, Dr. F. S. Dodds welcomed the Association with a very well-timed address, after which William Brown, Mayor of the city, offered the hospitality of the city, and thanked the Association for the honor conferred upon the city by selecting it as their place of meeting. Twenty-eight applications for membership were received and favorably acted upon. Several able and interesting papers on obstetrics were read and discussed, many members taking part. At the evening session an able and interesting public address on medical topics, by Dr. Barcly, of Marion, was delivered in the presence of a large and intelligent audience.

